# Probiotics Regulate Gut Microbiota: An Effective Method to Improve Immunity

**DOI:** 10.3390/molecules26196076

**Published:** 2021-10-08

**Authors:** Xinzhou Wang, Peng Zhang, Xin Zhang

**Affiliations:** 1 Department of Food Science and Engineering, Ningbo University, Ningbo 315211, China; wangxinzhounbu@163.com; 2 Department of Student Affairs, Xinyang Normal University, Xinyang 464000, China

**Keywords:** gut microbiota, probiotics, immunity

## Abstract

Probiotics are beneficial active microorganisms that colonize the human intestines and change the composition of the flora in particular parts of the host. Recently, the use of probiotics to regulate intestinal flora to improve host immunity has received widespread attention. Recent evidence has shown that probiotics play significant roles in gut microbiota composition, which can inhibit the colonization of pathogenic bacteria in the intestine, help the host build a healthy intestinal mucosa protective layer, and enhance the host immune system. Based on the close relationship between the gut microbiota and human immunity, it has become an extremely effective way to improve human immunity by regulating the gut microbiome with probiotics. In this review, we discussed the influence of probiotics on the gut microbiota and human immunity, and the relationship between immunity, probiotics, gut microbiota, and life quality. We further emphasized the regulation of gut microflora through probiotics, thereby enhancing human immunity and improving people’s lives.

## 1. Introduction

At the beginning of the 20th century, Nobel Prize winner Elie Metchnikoff proposed the concept of probiotics (meaning “for life”) [1] and the modern definition of probiotics as “live microorganisms, when administered enough confers the health of the host”. Probiotics mainly exist in the human intestines and can play a beneficial role in the host by maintaining the balance of intestinal microbes [2]. In daily life, common probiotics, such as *Lactobacillus* or *Bifidobacterium*, are usually consumed as active bacteria preparations [3]. In the past few years, research on probiotics has made significant progress, and a large number of studies have proven that probiotics play an essential role in maintaining human health. For example, probiotics can play a role in the treatment of chronic inflammatory diseases, for example, Crohn’s disease. In addition, probiotics can also have an anti-cancer, anti-obesity, and anti-diabetes effect [3]. There is growing evidence that probiotics can boost immunity and maintain health.

In recent years, there have been two critical signs of progress in intestinal microbiology and immunology. First, it has been demonstrated that the gut microbes primarily modulate the internal host environment. Second, the composition and products of intestinal microorganisms have a great influence on the immune response [3,4]. Accordingly, regulation of intestinal microorganisms has become a new method to maintain health and improve immunity, which has been widely focused on. There is increasing evidence that gut microbes play an essential role in the immune system. For example, short-chain fatty acids (SCFAs) produced by intestinal microorganisms can enhance the epithelial barrier function and reach other organs, acting on antigen-presenting cells, and reducing inflammation in related diseases [5]. Moreover, studies have shown that intestinal microflora can also affect immune function outside the intestine. For example, mice lacking a single G protein-coupled receptor, GPR43, have a profoundly altered inflammatory response [4]. Further study on the interaction between intestinal microorganisms and human immunity will help to prevent diseases and maintain human health.

As we all know, the human immune system can be divided into two branches—innate and adaptive immunity, respectively. First, compared to adaptive immunity, the function of innate immunity does not need to be regulated through acquired development, but as an initial immune barrier against foreign and harmful substances [6]. Second, adaptive immunity has a more refined recognition repertoire through growth and development, allowing more precise recognition of self- and non-self-antigens. Adaptive immunity comprises tightly regulated interactions between T and B lymphocytes and antigen-presenting cells, which promote immune memory generation, host immune homeostasis regulation, and pathogen-specific immune effects [7]. Studies have shown that gut microbiota is closely related to innate immunity and adaptive immunity. For example, the experiment results confirmed that flagellin specifically elicits an adaptive immune response, guided by innate immunity, and can regulate flagella production by the microbiota, which helps maintain the integrity of the mucosal barrier and homeostasis [8]. Additionally, the data showed that the adaptive immune response is closely related to intestinal microbiota composition, which can play a role in resisting severe diseases [9]. Overall, the role of the gut microbiota in shaping the complete immune system has become indisputable [10].

In general, probiotics are considered to be dietary factors that can influence the human gut microbiota and also have a regulatory effect on the composition and structure of the intestinal flora. It should be emphasized that the influence of intestinal flora on the immune system is vast. In the human body, intestinal flora can maintain the integrity of the mucosal barrier, provide nutrients, and resist pathogens [11]. Interestingly, recent research has found that the immune system plays a role in the relationship between sleep and skin character [12]. In addition, the central nervous system (CNS) is also affected by the immune system [13]. Accordingly, immunity directly affects people’s lives, and using probiotics to regulate intestinal bacteria has become an effective way to improve immunity. This review discusses the influence of probiotics on the gut microbiota and human immunity, and the relationship between immunity and life quality. Furthermore, the strategies of using probiotics to enhance immunity are highlighted.

## 2. The Mechanisms of Probiotics to Exert Their Beneficial Effects

With the development of probiotics research, the mechanism of how probiotics maintain health is becoming more apparent. In recent years, the most notable part has been preventing pathogens from adhering to the intestinal surface, maintaining the epithelial barrier, and modulating the immune system [14]. In addition, probiotics also have a certain regulatory effect on intestinal microecology and play a beneficial role in the human body.

### 2.1. Regulation of Intestinal Flora by Probiotics

As research progresses, the regulation of intestinal flora is effective in boosting immunity, treating metabolic disorders, and even treating mental illnesses [15]. As a class of beneficial microorganisms, probiotics also play a massive role in regulating intestinal flora [2]. According to Bagarolli et al. [16], mice fed a high-fat diet showed significant alterations in the intestinal flora associated with a range of diseases. Injecting probiotics into obese animals revealed that the Firmicutes of the intestinal flora decreased and the Actinobacteria increased. Treatment with probiotics can reverse intestinal flora dysbiosis and treat inflammatory responses in mice. Intestinal flora is a critical point in treating inflammatory diseases. We can infer that the regulation of intestinal flora by probiotics can effectively treat related inflammatory disorders. Researchers found that compared to the control group, the beneficial bacteria (such as Oscillibacter and Prevotella) and the concentrations of SCFAs in the intestinal flora of the probiotic-treated mice increased significantly [17]. SCFAs are of great research value as important metabolites of intestinal microorganisms. Today, SCFAs can enhance immunity [18] and act as a chemical signal for brain–gut communication [19]. By using probiotics, the number of SCFAs producing microorganisms in the gut can be increased. By doing so, this can have a beneficial effect on our immune system and maintain a healthy lifestyle. In addition, a large number of experiments have confirmed the regulatory effect of probiotics on intestinal flora, which is an indisputable fact. For example, experiments by Oh et al. [20] noted that probiotics could improve the decline in intestinal flora diversity caused by the use of antibiotics. In another trial, after patients with inflammatory bowel disease (IBD) consumed yogurt, probiotics such as Bifidobacterium and Lactobacillus in the patients’ intestines increased, which helped improve intestinal function [21]. There is no doubt that probiotics have a regulatory effect on the intestinal flora and have great potential in treating various diseases in the future.

### 2.2. Maintenance of the Epithelial Barrier

**Figure 1:**
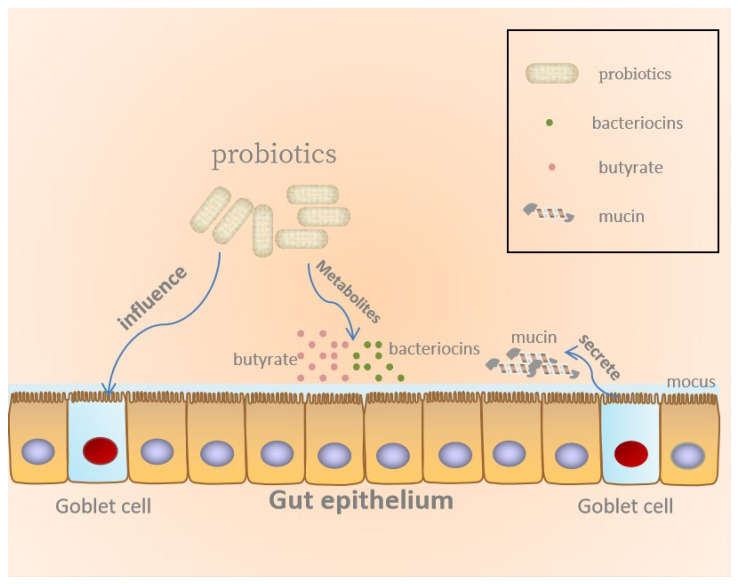
Probiotics maintain gut epithelial barrier.

There is substantial evidence that probiotics can strengthen the intestinal barrier, regulate mucosal immune function, and produce metabolites beneficial to the host to maintain health, as illustrated in Figure 1 [22,23]. The largest mucosal surface of the human body is the intestinal epithelium, which is composed of a single layer of intestinal epithelial cells (IECs) organized into villi and crypts [24]. One of the intestinal epithelium functions is to establish a physical and biochemical barrier between the external environment and the host immune system [23,25]. The mucosal barrier is the first drug-resistant barrier that pathogenic microorganisms encounter after they invade the intestine. The mucus layer, the epithelial lining of mucosal tissues, as well as the immune cells in the subepithelial layer are all components of the mucosal barrier. Pathogens must cross the mucosal barrier before they reach the epithelium. It can be inferred that mucus content and structural changes could affect barrier function. Studies have found that harmful microorganisms in the gut can produce certain specific substances to degrade mucus, thereby harming human health [26]. The research proved that probiotics can refine the barrier function by promoting the secretion of mucus. For instance, the probiotic *L*. *plantarum* BMCM12 can secrete extracellular proteins, weaken the adhesion of pathogens, and protect the intestinal barrier [27]. Moreover, another function of probiotics is to guarantee the integrity of the epithelial barrier. Through the study of mice and Caco-2 intestinal cells, the researchers found that the probiotics mixture VSL#3 or L. rhamnosus GG (LGG) could directly interact with IECs and maintain the integrity of the epithelial barrier [28,29,30]. In addition to the direct effect of probiotics, the metabolites of probiotics can also act on the intestinal epithelial barrier. It is found that butyric acid, a metabolite of probiotics, motivates O_2_ consumption in the intestinal epithelium, thereby increasing the expression of barrier protective hypoxia-inducible factor (HIF) target genes and maintaining HIF stability [27]. Moreover, previous research works demonstrated that probiotics could reduce intestinal permeability to protect the human gut. For example, Caballero-Franco et al. [26] showed that probiotics can prompt goblet cells to produce mucin to inhibit pathogen adherence. General speaking, probiotics maintain intestinal permeability, mucosal barrier integrity and strengthen physical barriers to maintaining the host’s health.

### 2.3. Inhibition of Pathogen by Probiotics

There are a large number of microorganisms in the human intestine, including pathogenic microorganisms and probiotics. The homeostasis of the gut microbiota may be altered by pathogenic microorganisms, which can elevate the risk of developing related diseases [31]. In past studies, it has been well documented that probiotics can protect the intestinal tract by inhibiting pathogenic bacteria. Therefore, the effects of probiotics on inhibiting pathogens in the gut and their underlying mechanisms have received significant attention from the research community. There are many mechanisms for probiotics to inhibit pathogenic microorganisms, such as stimulation of epithelial barrier function, producing antimicrobial substances, limiting access of pathogenic microorganisms to nutrient resources, and competitive exclusion by competition for binding sites [32,33]. Conversely, a crucial beneficial mechanism of probiotics is the competitive exclusion of pathogens [30]. For example, the experiment by Fang et al. [34] showed that probiotic Escherichia coli Nissle 1917 (EcN) can secrete DegP (a bifunctional periplasmic protein) to inhibit enterohemorrhagic E. coli (EHEC). The probiotic Escherichia coli outcompetes pathogenic biofilms via extracellular DegP activity during dual-species biofilm formation. In another experiment, probiotics can secrete antibacterial substances, causing steric hindrance and competitive adhesion sites and nutrients to prevent Helicobacter pylori from binding to epithelial cells [35]. On the other hand, secreting antibacterial compounds is another vital function of probiotics. A typical example is that probiotics can secrete organic acids during carbohydrate fermentation, such as butyric acid, acetic acid, and propionic acid. Organic acids have been considered to be the main antimicrobial compounds responsible for their inhibitory activity against pathogens. The decrease in pH and the presence of *undissociated* acid make organic acids have a certain antibacterial activity [30]. Interestingly, some experiments also found that through inhibition of the pathogen signal system, intestinal pathogens can be eliminated. The investigation by Piewngam et al. [36] showed that the inhibitory effect of Bacillus isolates on S. aureus colonization is due to a secreted substance that inhibits Agr signaling. As time goes on, the ways probiotics can inhibit pathogenic microorganisms will become more abundant.

### 2.4. The Modulation and Proper Maturation of the Immune System

Recent research studies have suggested that live probiotics or their metabolites could interact with diverse immune cells and confer them to have immunoregulatory functions [25,37,38]. To sum up, the effects of probiotics on the immune system are shown in the following aspects.

Probiotics have a certain influence on IECs. Bacterial fragments of probiotics can be internalized into IECs, activate related immune cells, and promote IECs to start a complex signal network. Its main function is to activate the innate response and the cytokines released by T cells and stimulate immune cells related to lamina propria. The results showed that probiotics could activate the immune system [23,37].

Probiotics promote the maturation of humoral immune mechanism. Probiotics entering the intestine can stimulate the production of the IgA antibody [39]. Studies have found that oral probiotics can effectively increase IgA^+^ cells in intestinal lamina propria. Many studies have shown that probiotics can induce the IgA cycle, strengthen and maintain the immune surveillance of the mucosa away from the intestine, and promote the maturation of the humoral immune mechanism [23,28,40].

Probiotics can increase the number of macrophages and dendritic cells (DCs) in lamina propria and enhance their function in the intestine for a period of time. DCs are mainly responsible for recognizing and eliminating exogenous pathogens in the immune system, and are the primary cell type involved as “sensors” of microbial ligands through activation of innate immune receptors. DCs can enter the mucosa-associated lymphoid tissue or discharge lymph nodes continuously through the antigen barrier [23,40,41,42]. Macrophages are also important immune cells. Macrophages are mainly responsible for phagocytosis of cell debris and pathogens, the activation of lymphocytes and other immune cells against pathogens, and the fixation of free cells. Recently, there are many studies on the interaction between probiotics and macrophages. Among them, one study showed that *Lactobacillus* probiotic strains activated the in vitro inflammatory response of macrophages via the synthesis of proinflammatory mediators, including cytokines, reactive oxygen species (ROS), and participation in the signaling cascades, such as the nuclear factor kB (NF-kB) and Toll-like receptor 2 (TLR2) pathways [39].

Nowadays, the regulation of probiotics on the immune system is used to prevent some common diseases [43]. The ability of probiotics to modulate the immune system needs to be further explored.

## 3. Effect of Gut Microbiome on Immunity

Focus on the beneficial effects of intestinal microorganisms on the host has gradually increased. Recently, according to research results, intestinal microorganisms have certain effects on various host functions, including the host’s immune system [10]. Indeed, the gut microbiota has been found to have a wide range of effects on inflammatory/autoimmune disease, such as allergy and cancer [44], and when the homeostasis of the host and its intestinal microorganisms is broken, the intestinal flora will cause some diseases [45]. Therefore, to explore the role of intestinal microbiota in the immune system and to study the treatment and prevention of related diseases by intestinal microbiome are highly significant.

### 3.1. The Role of Gut Microbiome in the Immune System

There are many kinds of microorganisms in the intestine, some of which mutually interact with the host [46]. These commensal microbes in the gut have a major impact on the immune system, such as the prevention of pathogen attachment and polarization of intestinal-specific immune response [45]. In general, the intestinal microbiome is closely related to host immunity and plays an important role in the immune system [47].

Recently, researchers have proven that intestinal flora plays an essential role in the immune system through various methods. Among them, germ-free (GF) models that reveal the effects of the gut microbiota on the immune system have become the most common approach [47]. Until now, our understanding of the relationship between gut microbiota and the immune system has primarily been informed by the studies of GF mice, which have maintained a lack of microflora in the gastrointestinal tract since birth. By supplementing GF mice with specific microorganisms, including pathogenic microorganisms and symbiotic microorganisms, we have a detailed understanding of the interaction between different microorganisms and the host immune system [48,49]. For instance, the experiment by Lamouse-Smith et al. [50] chose GF mice and untreated control mice as subjects. They subcutaneously immunized 12-week-old GF and conventional untreated control mice with ovalbumin (Ova) and completed Freund’s adjuvant. The researchers found that the ova-specific antibody titers of untreated control mice were significantly higher than those of GF mice, which meant that in the absence of intestinal flora, mice exhibited defects in the development of immune cells and immune tissues. Additionally, usually, antibodies cannot be produced after a systemic vaccination. With the development of research, thanks to GF models, the protective effect of intestinal microorganisms on the host has been further confirmed. The experiment showed that under identical culture conditions, GF animals display a wide range of defects in developing gut-associated lymphoid tissues (GALTs) compared with conventional animals. Moreover, GF animals show an absent mucous layer and altered IgA secretion [10,24]. Based on several previous experiments, we can further analyze the role of intestinal microorganisms concerning the immune system. The intestinal microorganisms lodged in the gut are closely associated with the epithelial cells, which are an essential part of the intestinal immune system. The receptors on the epithelial cells receive signals from the intestinal microbes and respond in a certain way to ensure homeostasis in the intestine [45,46]. Researchers found that the development of GALTs also depended on gut microbes [44]. GALTs serve as important immune structures with many vital functions. For example, antigen-presenting cells can absorb and present antigens in this structure, which helps to protect the body from infection. However, it has been found that GALTs are not fully developed in GF mice, presumably due to a lack of gut microbes [45,47]. In addition, intestinal microorganisms are closely associated with a variety of immune cells, typically T helper 17 (T_H_17) cells, regulatory T (T_Reg_) cells, and gut-specific B cells [45]. We can consider this point further. In addition, we may purposely regulate the intestinal flora to strengthen the immune system for the treatment of related diseases, such as the prevention of cancer, the treatment of depression, etc.

As is known, the gut has an immune system that can prevent the invasion of pathogenic microorganisms and create a suitable environment for beneficial bacteria, which plays a crucial role in maintaining host health. Many studies have proven that the intestinal microbiome can strengthen the intestinal immune system [25,42], indicating intestinal flora plays an important role in the immune system.

### 3.2. Gut Microbiome, Food Allergy, Cancer and Depression

Allergy is one of the most common chronic diseases worldwide and is often associated with hyper-activation of the T helper 2 (Th2) arm of adaptive immunity [51]. Food allergy is the most common; an allergy could be triggered by virtually any food, and the “major allergens” include egg, nuts, wheat, fish, and milk [52]. Food allergy affects many people all over the world. Food allergy is defined as an adverse health effect arising from a specific immune response that occurs reproducibly on exposure to a given food [53] and can be either non-IgE mediated or IgE-mediated [54,55]. The study confirmed that in multiple interconnected networks of host immunity and homeostasis, the gut microbiota plays an important role in maintaining homeostasis [56]. The gut microbiota is involved in structural modification of the host intestinal mucosa, neurotransmission, vitamin K production, as well as the development of immune responses [57]. Currently, many experiments have suggested a role for the gut microbiota in the pathogenesis and progression of food allergy [53], and gut microbes can function to maintain the immune system’s efficiency [58]. A study of the early life gut microbiome of children with egg allergy found that the early life gut microbiome of normal children had lower diversity and distinct taxa than children with egg allergy. Moreover, this experiment also found that Lachnospiraceae and Ruminococcaceae are more enriched in the gut of children allergic to eggs [59]. More information about the relationship between the intestinal microbiome and food allergy was obtained by using GF mice. Using broad-spectrum antibiotics to treat both GF mice and mice, it was found that the serum IgE levels and numbers of basophils of GF mice were increased. The experimental results suggested that the host required the gut microbiota to regulate IgE and basophil-mediated responses associated with food allergy [60]. Another experiment further showed by studying GF mouse models that the loss of gut flora could induce incomplete development of GALTs, leading to a Th2 skewed immune response [61]. There is a mountain of evidence that the gut microbiome is closely related to food allergy.

The origin of cancer is a malignant tumor of epithelial tissue; the generation of cancer is generally thought to be secondary to a state of local chronic inflammation [62]. The occurrence of cancer is directly related to immune deficiency. With the discovery of the beneficial effect of intestinal flora on immunity, the relationship between the intestinal microbiome and cancer has been paid more and more attention [63]. The most typical example is the relationship between intestinal flora and colorectal cancer. Colorectal cancer has a high incidence and mortality [64,65]. Through research in recent years, it has been proven that there is a close relationship between intestinal microorganisms and colorectal cancer. The study found that specific species of bacteria may affect both the risk of colorectal cancer and the growth of existing tumors [66]. For instance, the experiment by Ahn et al. [67] showed that microbial diversity was significantly lower in the gut of patients with colorectal cancer. More specifically, colorectal cancer patients had higher numbers of Fusobacterium and Porphyromonas and a lower relative abundance of clostridia. This result has also been confirmed in other experiments; other studies have found that survival of patients with colorectal cancer is associated with the concentration of Fusobacterium nucleatum [64]. In animal models, the researchers found that gut microbes can produce corresponding harmful metabolites to act on the host’s immune system, inducing the release of genotoxic virulence factors and promoting colorectal carcinogenesis [65,68]. Analysis of the stools of colorectal cancer patients revealed a significant dysbiosis of the intestinal flora, as evidenced by a decrease in the number of butyric acid-producing commensal bacteria and an increase in the number of harmful pro-inflammatory opportunistic pathogens [65]. As mentioned in the above paragraph, the intestinal epithelial barrier is an important part of the immune system and is closely related to intestinal microorganisms. Dysbiosis can damage the intestinal epithelial barrier and then increase the number of inflammatory cells. The inflammatory cells may cause overexpression of cytokines and chemokines, which have been detected in human colorectal cancer [65,66]. The metabolites of intestinal microorganisms are another important factor affecting the occurrence of colorectal cancer. SCFAs are a special class of metabolites produced by intestinal microorganisms. SCFAs can modulate inflammation, epithelial proliferation, and apoptosis. SCFAs can also improve immunity and reduce the risk of developing colorectal cancer. Conversely, microbial metabolites can lead to inflammatory responses and epithelial hyperproliferation that predispose to tumorigenesis [59]. It has been proven by many experiments that the gut microbiota is an important determinant of colon tumor susceptibility.

The common beneficial microorganisms in the gut include Bifidobacterium sp., Streptococcus thermophilus, Lactobacillus sp., and Saccharomyces boulardii [69]. They have become a regular part of our dietary habits [70]. The gut microbiome may influence normal brain development, mood, and pain sensitivity. Moreover, several research findings have shown that probiotics can affect the CNS by regulating the intestinal flora and preventing mental diseases through the gut–brain axis [71,72,73]. Recently, it has been proven that these beneficial microorganisms in the intestine can enhance immunity, and the improvement of immunity helps prevent the occurrence of depression. Depression is the most common disorder. The main clinical manifestations are depression, slow thinking, and impaired cognitive function. With the continuous development of science and technology, metagenomics and molecular tools have been further improved, contributing to the continued promotion of research [74]. In studies of depressed patients, it was found that cell-mediated activation of adaptive immunity appears to be dramatically different in depressed patients compared to the general population [75]. Moreover, in past studies, there have been a large number of experimental results that have proven that the activation of innate immune mechanisms, such as the activation of the proinflammatory cytokines interleukin-6 and interleukin-1, has a certain association with the development of depression [76]. The link between the occurrence of depression and the immune system is obvious. The intestinal flora has the function of enhancing immunity and refining the immune system, which has a certain theoretical basis for advocating that intestinal flora can improve immunity to treat depression.

## 4. Probiotics, Gut Microbiome, Immunity and People’s Life

The importance of intestinal flora for the immune system and the role of probiotics in improving immunity have been confirmed. However, what benefits can improved immunity bring to people’s lives? In the following, we will introduce several examples of improving immunity through probiotics.

### 4.1. The Role of Gut Microbiome in Obesity and Local Inflammation of Adipose Tissue

Obesity is a common metabolic disease. There are many factors that cause obesity. In addition to lifestyle, diet, and genetic factors, some studies have suggested that a cause of obesity could also be disorders of the intestinal flora [77,78,79]. Moreover, patients with obesity are more likely to have local inflammation in adipose tissue than healthy lean individuals, and then, the local inflammation can turn into systemic inflammation [80]. Intestinal flora can prevent local inflammation of adipose tissue by preventing obesity and enhancing immunity. It can be seen from the above that there is an interactive relationship between intestinal flora and the immune system, and the intestinal flora plays an important role in the immune system. In addition, it is well established that the gut microbiota can influence the development of obesity. Using genetic sequencing of fecal samples from multiple obese patients, researchers identified different strains among them and compared them with lean volunteers. They found that obese individuals had significantly fewer Bacteroidetes and more Firmicutes [81], which means the incidence of obesity may be related to the proportion of Firmicutes and Bacteroidetes [82]. In another landmark experiment, the researchers introduced normal cecal microbiota to adult GF mice. Two weeks later, despite a reduction in food intake, adult GF mice maintained a 60% increase in body insulin resistance and fat [83]. This proves that intestinal flora has a significant role in preventing obesity. In conclusion, intestinal flora has a considerable effect on preventing local inflammation of adipose tissue.

### 4.2. The Relationship between Immunity and Sleep and the Effect of Gut Microbiome on Sleep

**Figure 2:**
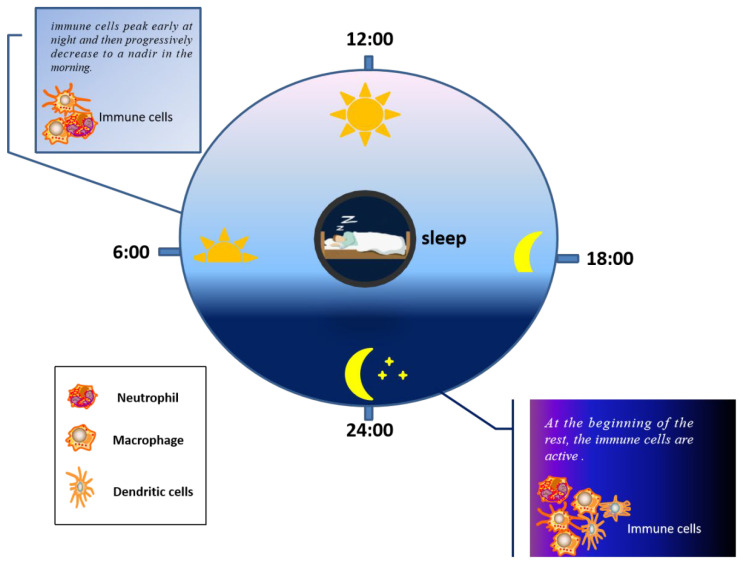
Activity of immune cells at different times of sleep.

The importance of sleep for health is self-evident. As is known, sleep loss can cause some diseases. Indeed, many people sleep as an aid for recuperation from disease states [84]. Thousands of years ago, people began to explore the secret between sleep and health [85]. In recent decades, the relationship between immunity and sleep has been proposed. Nowadays, experimentally, the impact of rest and the immune system is reciprocal [12]. Sleep has been shown to regulate inflammatory processes. An experiment to determine circadian rhythms by multiple measurements over 24 h found that when poor sleep quality interferes with the normal circadian rhythm, the increase in IL-6 during sleep is promoted, and growth is reduced by one and a half by complete deprivation of nocturnal sleep [86]. In another experiment, during the normal sleep–wake cycle, the immune system showed a dynamic change over this time. The number of immune cells reached a peak at night and returned to their lowest in the morning [12]; more specifically, new immune cells, such as macrophages, DC and neutrophils, peak during the early rest period [87], as illustrated in Figure 2. Moreover, studies have found that the decline in immunity caused by sleep deprivation also increases the risk of infectious diseases, for instance, influenza. In one experiment, subjects were inoculated with a known amount of rhinoviruses, and the volunteers’ sleep was continuously controlled and monitored for 5 to 7 days. The researchers found that shortening sleep time and reducing sleep quality would increase the risk of catching a cold [88]. To show more clearly the impact of sleep on the persistence of immune response, vaccination has become an ideal experimental model. Take the immune response obtained after influenza vaccination as an example. Subjects were divided into two groups: those who sleep 4 h a day and those who sleep 8 h a day. Both groups were vaccinated against influenza. The results showed that the concentration of influenza virus-specific antibody measured ten days after vaccination was twice that of subjects who slept 8 h a day compared to what was measured in subjects who slept 4 h a day [88]. A similar result was found in another experiment. After H1N1 vaccination, if there is no sleep at night, antigen-specific antibody response could be reduced [89]. All this evidence proved the close relationship between immunity and sleep. We can take some measures, such as adjusting our daily diet or taking probiotics to purposefully regulate the composition of our intestinal microorganisms, to enhance immunity and, ultimately, improve the quality of our sleep.

In recent years, studies have shown that sleep is closely related to the diversity of the intestinal flora. For example, Smith et al. [90] demonstrated that the diversity of the gut microbiota would decrease because of sleep fragmentation, but increase due to good sleep quality and enough sleep time. They also found that short-term sleep control has little effect on intestinal microbial diversity, but intestinal microbial diversity can have a long-term impact on sleep quality. In summary, the experiment concluded that the diversity of the gut microbiome promotes healthier sleep. Moreover, another study found that transplanting fecal microbes to regulate gut microbes can improve sleep efficiency. This experiment has shown that the regulation of intestinal flora may lead to novel sleep intervention strategies [90]. Conversely, sleep can also affect intestinal flora. A study in mice found shifts in the microbiome after longer-term sleep fragmentation [91]. More specifically, the treatment with *Lactobacillus casei* strain Shirota (LcS) causes a positive impact on patients’ sleep duration and quality of sleep [92]. These results all proved that there is a close relationship between sleep and intestinal flora.

### 4.3. The Relationship between Intestinal Flora and Skin and the Use of Probiotics to Improve Skin Quality

Up to now, there have been many experiments demonstrating a bidirectional link between the skin and the gut, and the character of some gastrointestinal disorders can be manifested through the skin. With the continuous research on the relationship between intestinal microorganisms and host health, scientists are now investigating how local microbes influence the immune competence of distant organs. Among them, the most focus has been on how gut microbes affect lung, heart, skin and other organs [93]. Therefore, researchers found that the intestinal microbiome is closely related to common skin diseases, such as acne, psoriasis, and atopic dermatitis (AD) [94]. Moreover, in addition to some traditional methods, the use of probiotics in treating these diseases has been paid more and more attention. Take the relationship between intestinal microbiome and AD as an example. AD is a common chronic inflammatory skin disease in the world [95]. Nowadays, the mainstay of AD treatment is to use anti-inflammatory and emollients, by which the disadvantages of poor immune tolerance and barrier dysfunction are compensated. In recent years, studies have found that probiotics can play a role in preventing and treating AD by enhancing immunity [94]. For example, one study in Norway found that the incidence rate of AD can be effectively reduced by supplying probiotic milk to women and infants before and after delivery [94]. In another study, it was found that compared with the healthy control group, the level of *Bifidobacterium* in the intestinal tract of AD patients was lower, and the level of *Bifidobacterium* in the intestinal tract was negatively correlated with the severity of disease in AD patients. The study also showed that the changing of intestinal flora may be earlier than the development of AD. Accordingly, we can infer that intestinal flora disorders may be one of the reasons for the occurrence of AD [95].

Another common skin disease is acne. Acne is a kind of chronic skin disease, mainly caused by keratin changes, inflammation, hormone-induced hyper seborrhea, and decreased immunity. Common sites of occurrence include the neck, face, as well as back [96]. The most common treatments include topical and oral retinoids, benzoyl peroxide, antimicrobial agents, as well as a proper skincare routine [97]. In recent years, researchers have found that probiotics also play a role in the treatment of acne. Studies have confirmed that probiotics, such as *Lactococcus* sp. HY 449, can directly inhibit the occurrence of *P. acnes* through the production of antimicrobial proteins [43]. In an experiment, researchers found that after ingestion of probiotic tablets of *Lactobacillus bulgaricus* and *Lactobacillus acidophilus* by multiple acne patients who participated in the experiment, it was found that the majority of the acne patients’ conditions improved [94]. Through these years of research on the brain–gut–skin axis, it has been found that oral probiotics are very helpful to improve skin therapy [43]. Through an animal study, it was found that an oral solution containing the probiotic *L. reuteri* significantly limited the major histocompatible cells around the hair follicles compared to the control group [43].

Psoriasis, like acne, is a form of chronic inflammatory skin disease that affects many people worldwide [98]. Psoriasis usually presents as erythematous or thick scaly plaques on the skin [99]. In recent studies, psoriasis has been associated with the gut microbiome. In a study by Hidalgo-Cantabrana et al. [100], they found that the gut flora of patients with psoriasis was severely dysregulated, the diversity of certain bacterial taxa reduced, and the abundance altered. The number of *Firmicutes* and *Actinobacteria* in the gut microbiota of patients with psoriasis increased significantly, but *Proteobacteria* and *Bacteroides* decreased. The same results were obtained in the research carried out by Scher et al. [101]. The study found that the abundance of *Actinobacteria* in the gut of patients with psoriasis was less than that of healthy controls. Masallat et al. [102] found that the prevalence of psoriasis was negatively correlated with the concentration of actinomycetes by the Psoriasis Activity and Severity Index (PASI) score. An increased *Firmicutes*/*Bacteroidetes* ratio was also found in patients with psoriasis. In summary, it can be assumed that abnormal changes in the number of different types of bacteria in the intestinal tract caused by intestinal microbial dysbiosis are one of the causes of the development of psoriasis [103]. In recent years, although there has been limited evidence for the use of probiotic products in the treatment of psoriasis, research is still progressing. It is reported that oral administration of *Lactobacillus pentosus* GMNL-77 could effectively treat skin inflammation due to imiquimod treatment in mice. The researchers further found that *Lactobacillus pentosus* GMNL-77 also ameliorated erythematous scaling lesions in psoriatic mice due to imiquimod treatment [104]. This experiment provided a theoretical basis for probiotic products to improve psoriasis. Another study found that psoriasis is closely related to the dysfunction of intestinal barrier function, and probiotics have a certain protective effect on the intestinal barrier. Therefore, regular supplementation of probiotics is an effective way to improve skin quality. It does not cost much and is very safe [105].

In a word, a large number of experiments confirmed the close relationship between intestinal flora and skin quality. With the continuous progress of research and the traditional treatment of common skin diseases, probiotics will become another effective treatment. People are paying more and more attention to intestinal flora and probiotics in the direction of improving skin quality.

## 5. Conclusions

As a kind of beneficial microorganism, probiotics can regulate the composition of intestinal flora and enhance immunity. Probiotics can improve host immunity by maintaining the epithelial barrier, inhibiting pathogens from adhering to the intestinal surface, and modulating and properly maturing the immune system. Moreover, probiotics can also improve host immunity by affecting intestinal flora to treat certain diseases. Nowadays, it has been proven that there is a close relationship between probiotics, intestinal flora and immunity. In the future, the mechanism by which probiotics regulate the structure of intestinal flora and improve immunity will be further elucidated, and will also be an effective way to improve people’s quality of life.
